# NOTIFICATION: Regarding Zhou et al. (DOI: 10.1111/cns.14137) and O'Donnell (DOI: 10.1111/cns.14541)

**DOI:** 10.1002/cns.71084

**Published:** 2026-08-20

Following publication of the article by Zhou et al. [1], the journal received correspondence by John C. O'Donnell raising concerns regarding the interpretation and terminology used in the study, particularly in relation to the use of the term “Disorders of Consciousness” and the translational implications of the experimental model, which resulted in the publication of a Letter to the Editor [2].

The editorial team evaluated the issues raised through editorial assessment and external consultation. While differing scientific opinions remain regarding the limitations and interpretation of the model, no evidence of scientific misconduct, fabrication, or falsification was identified.

The editors consider this matter to reflect an ongoing scientific and conceptual discussion concerning the interpretation and translational relevance of the model rather than a research integrity issue.

Readers are encouraged to consider the published correspondence and the linked subsequent discussion tagged as “Hot Topic Discussion” in CNS Neuroscience & Therapeutics.

The Editorial Team

*CNS Neuroscience & Therapeutics*
